# Loss of Heterozygosity Is Present in *SEC63* Germline Carriers with Polycystic Liver Disease

**DOI:** 10.1371/journal.pone.0050324

**Published:** 2012-11-28

**Authors:** Manoe J. Janssen, Jody Salomon, René H. M. te Morsche, Joost P. H. Drenth

**Affiliations:** Department of Gastroenterology and Hepatology, Radboud University Nijmegen Medical Centre, Nijmegen, The Netherlands; Instituto de Ciencia de Materiales de Madrid - Instituto de Biomedicina de Valencia, Spain

## Abstract

Polycystic liver disease (PCLD) is an autosomal dominant disorder characterised by multiple fluid filled cysts in the liver. This rare disease is caused by heterozygous germline mutations in *PRKCSH* and *SEC63*. We previously found that, in patients with a *PRKCSH* mutation, over 76% of the cysts acquired a somatic ‘second-hit’ mutation in the wild type *PRKCSH* allele. We hypothesise that somatic second-hit mutations are a general mechanism of cyst formation in PCLD which also plays a role in PCLD patients carrying a *SEC63* germline mutation. We collected cyst epithelial cells from 52 liver cysts from three different *SEC63* patients using laser microdissection. DNA samples were sequenced to identify loss of heterozygosity (LOH) mutations and other somatic mutations in cyst epithelial DNA. We discovered somatic *SEC63* mutations in patient 3 (1/14 cysts), but not in patient 1 and 2 (38 cysts). Upon review we found that the germline mutation of patient 1 and 2 (*SEC63* c.1703_1705delAAG) was present in the same frequency in DNA samples from healthy controls, suggesting that this variant is not causative of PCLD. In conclusion, as somatic second-hit mutations also play a role in cyst formation in patients with a *SEC63* germline mutation, this appears to be a general mechanism of cyst formation in PCLD.

## Introduction

Polycystic liver disease (PCLD; MIM# 174050) is a rare, dominantly inherited, disorder characterised by multiple fluid filled cysts in the liver. The cysts, which develop from bile duct epithelial cells (cholangiocytes), increase in size and number over time and can lead to a massive increase in liver volume [1]. So far two genes, *PRKCSH* (MIM# 177060) [2], [3] and *SEC63* (MIM# 608648) [4], have been associated with the development of PCLD. A systematic sequencing effort demonstrated that *PRKCSH* mutations account for 15% of the PCLD cases, whereas *SEC63* mutations can be found in 6% [5].

In the case of a dominant disorder it is not always clear how the heterozygous mutations can lead to disease and which mechanisms are involved. The mutations can either lead to the production of a mutated protein which disrupts the cell function, or result in loss of functional gene product which can lead to misregulation of dosage dependent genes. Often, loss of a single allele will not have severe consequences for the function of the cell and only after the remaining allele is lost this will cause disease.

Recently, we demonstrated that in PCLD patients harbouring a heterozygous *PRKCSH* mutation, over 76% of the cysts acquired a somatic ‘second-hit’ mutation in *PRKCSH* [6]. These mutations lead to total loss of functional *PRKCSH* in the cyst epithelium suggesting that PCLD is recessive on a cellular level. This finding is consistent with reports on other cystic diseases, such as autosomal dominant polycystic kidney disease (ADPKD) [7]–[12], where somatic second-hit mutations also are present in cyst epithelia. We could also show that loss of the wild type allele corresponded to loss of *PRKCSH* gene product hepatocystin in these cells [6], [13]. Much less is known about how the heterozygous mutations in *SEC63* lead to cyst formation. On an immunohistological level, cysts from *PRKCSH* and *SEC63* patients show a different expression pattern for various proteins including MUC1 and C-erbB-2. Furthermore, no loss of SEC63 protein has been reported in cysts form patients with a germline mutation in this gene, which could reflect a different mechanism of cyst development in cysts from *SEC63* patients [14].

Although there is genetic and immunohistological heterogeneity among PCLD patients, the clinical presentation, the presence of a normal bile duct system and the focal growth of the cysts on the other hand, are features shared by all PCLD patients [15]. This suggests that the mechanism of cyst formation through second-hit mutations is similar among different genetic forms of PCLD. We therefore hypothesise that, similar to the situation in *PRKCSH*, somatic second-hit mutations are also an important step in cyst formation in patients with a *SEC63* germline mutation.

To this end we analysed 52 cyst samples from 3 patients carrying a *SEC63* mutation. In one cyst sample we found loss of heterozygosity (LOH), whereas we did not find any somatic changes in 38 samples derived from the other two patients. After reviewing the different germline mutations we found that somatic inactivation only occurred against the background of the severe truncating germline mutation.

These results show that somatic second-hit mutations play a role in cyst formation of both *PRKCSH* and *SEC63* mutation carriers.

## Materials and Methods

### Ethics Statement

Liver tissue and blood samples of patients were obtained and stored in the course of treatment following the Dutch Code for the proper secondary use of human tissue. Use of this tissue for research was reviewed and approved by the regional ethics review board “Commissie Mensgebonden Onderzoek (CMO) regio Arnhem-Nijmegen”.

### DNA and Tissue Samples

We used the following strategy to obtain all known liver cyst samples from *SEC63* germline mutation carriers within the Netherlands. Patients were selected based on sequencing results from our molecular diagnostic laboratory, which routinely performs diagnostic tests for PCLD. Each PCLD patient is tested for *PRKCSH* as well as *SEC63* and the database now holds 505 patients. Using this database we could readily identify 29 patients with a *SEC63* mutation. We cross checked these patients against the Dutch National Pathology database to find those patients who had undergone laparoscopic cyst fenestration because of severe symptoms [16] and for whom cyst tissue samples would be available. Following this strategy we identified 3 *SEC63* germline mutation carriers for whom blood and tissue samples were available.

All patients were female, and age at the time of surgery varied between 33 and 41 years-of-age. We used fresh tissue samples that had been snap-frozen immediately after excision and stored at −80°C until analysis (n = 38; 2 patients), or formalin-fixed paraffin-embedded liver tissue samples that had routinely been collected for pathological examination (n = 14; 1 patient). All samples were collected with appropriate ethics approval: written informed consent for the use of secondary tissue was obtained from all patients.

We collected whole blood from 1000 healthy controls (2000 chromosomes) which were recruited after advertisement in local papers from the same geographical region as our patients.

### Genotyping

We screened patient DNA from whole blood for germline mutations in *SEC63* and *PRKCSH* using direct sequencing as described previously [6]. In brief, DNA from whole blood was isolated using the PureGene DNA isolation kit (Gentra Systems, Minneapolis, Minnesota, USA) and stored at 4°C. Exons and flanking intronic sequences were amplified using polymerase chain reaction (PCR) with specific primers. The amplified fragments were purified (QIAEXII Gel Extraction Kit, Qiagen, Hilden, Germany) and sequenced with the BigDye terminator kit and ABI3730 capillary sequencer (Perkin Elmer Applied Biosystems, Boston, MA, USA). Names of *SEC63* mutations refer to the NM_007214.4 transcript according to the HGVS guidelines.

We screened genomic DNA samples of 1000 healthy controls for the *SEC63* c.1703_1705delAAG mutation using PCR with the forward primer 5′-TAGTGAAATTGTCATCGAGTCAG-3′ and the reverse primer 5′-CGAGCAAGCAAACAAATGAA-3′ followed by high resolution melting (HRM). Melting curves were obtained from 65°C to 95°C with a ramp rate of 0.1°C/10 seconds in the CFX96TM Real-Time PCR Detection System (Biorad Laboratories, Hercules, CA, USA) with EvaGreen (Biotium, Hayward, CA, USA) as fluorescent dye. Melting curves were analyzed using the Precision Melt software (Biorad). Abnormal curves were then sent for sequencing.

### Laser Microdissection

Tissue sections (10 µm), from frozen or formalin-fixed paraffin-embedded liver samples, were mounted on cross-linked PEN-membrane slides (Leica Microsystems GmbH, Wetzlar, Germany), stained with Mayers Hematoxylin (1 min) and rinsed in tap water. Paraffin embedded sections were deparaffinized using xylene and ethanol prior to hematoxylin nuclear stain. Specific isolation of the cyst epithelial cells (300 to 2000 cells/sample) was carried out using a Leica Laser Microdissection system (LMD 6000) equipped with an UV laser (Leica Microsystems GmbH) [17]. For each patient we dissected liver cells (hepatocytes and other non-cyst epithelial cells) to serve as a control sample.

### DNA Isolation from Dissected Cells

DNA from the dissected cells was isolated using the QIAamp DNA Micro kit (Qiagen) according to instructions and with the use of carrier RNA. To increase the DNA yield and quality from formalin fixed tissue samples we made the following adjustments: samples were digested at 56°C for two days with occasional agitation; proteinase K solution (>600 mAU/ml) was added in two steps (5 µl on day 1 and 5 µl on day 2); after digestion and addition of buffer ATL, samples were incubated at 90°C for 1 hr to promote reverse cross-linking of the DNA. To obtain enough material for the *SEC63* and *PRKCSH* sequencing, cyst epithelial DNA was amplified using a commercially available whole genome amplification kit (GenomePlex WGA, Sigma-Aldrich, Saint Louis, MO, USA) and purified (GenElute PCR Clean-Up Kit, Sigma-Aldrich) prior to analysis. Sample amplification was performed in duplicate to control for any mutations resulting from the whole genome amplification procedure.

### Somatic Mutation Analysis

We conducted the following analyses on DNA isolated from laser dissected samples:

1. *SEC63 LOH analysis*. The region of the germline mutation was amplified using specific primers for the *SEC63* c.958G>T mutation (forward 5′-TGAAAATTCCTGAGACCCTTG-3′ and reverse 5′-TGCTGCTTTCATCCCACTAA-3′) and the *SEC63* c.1703_1705delAAG mutation (forward 5′-TAGTGAAATTGTCATCGAGTCAG-3′ and reverse 5′-CGAGCAAGCAAACAAATGAA-3′) followed by sequencing to determine the heterozygosity state of the germline mutation in both cyst epithelia and control liver cell samples.
2. *SEC63 and PRKCSH sequencing*. All *SEC63* and *PRKCSH* coding exons and flanking intronic sequences were sequenced (as described for genotyping) on amplified DNA to detect somatic mutations in the cysts. DNA isolated from whole blood of the patient was used as reference sample.

### LOH Region Analysis

We used 5 heterozygous SNPs across chromosome 6 to analyse the heterozygosity state in cyst DNA using PCR with specific primers: rs2012025 (foward 5′-GGGCCAGCAGAAATAACTTG-3′, reverse 5′-GCTGTCGTTCTCATCATCCA-3′), rs13220047 (forward 5′-TGTAGGTAAGGGAGATGCAC-3′, reverse 5′-CCTACTGGACTCAGTGGTTT-3′), rs675117 (forward 5′-AGCCACAAGCTTTGGAATTG-3′, reverse 5′-TTTAAATGCACTCACCAGAATTG-3′), rs12210583 (forward 5′-GCCTCAGAACCCTGAGCTG-3′, reverse 5′-ACCAAGGCTGTATCGCAATC-3′) and rs10946279 (forward 5′-GCCGTGAGACAGCAAGTGT-3′, reverse 5′-GGTTCCATCCCCAAGTCTCT-3′), followed by sequencing.

### Immunohistochemistry

Tissue sections (4 µm) from formalin-fixed paraffin-embedded PCLD liver tissue were obtained together with tissue sections for laser micro dissection. Sections were mounted on SuperFrost Plus glass slides (Thermo Scientific Waltham, MA, USA) using a water bath and allowed to dry overnight at 37°C. Prior to the staining the tissue sections were deparaffinised with xylene and hydrated with alcohol and distilled water. Samples were microwave heated and boiled for 10 min in Na-Citrate buffer pH 6, cooled down to room temperature, washed in phosphate buffered saline (PBS) and incubated for 30 min with 1%BSA/PBS (Bovine Serum Albumin, Sigma-Aldrich). Endogenous Avidin/Biotin was blocked (Vector laboratories, Burlingame, CA, USA) followed by overnight incubation with 1∶400 rabit anti-SEC63 antibody (kind gift from Prof. Dr. Enno Hartmann, University of Lübeck, Germany), 1∶200 mouse anti-hepatocystin antibody (sc10774, Santa Cruz, CA, USA) or 1∶400 mouse anti-Cytokeratin 19 (MU246-UC, BioGenex, San Ramon, CA) in 1%BSA/PBS. For each staining we included a negative control in which the primary antibody was omitted. Endogenous peroxidase was blocked in 0,3% H_2_O_2_/PBS for 10 min after which samples were washed in PBS. Samples were incubated for 1 hr with secondary antibody (biotinylated anti-mouse or anti-rabit IgG, Vector laboratories), washed and bound to Horse-Radish-Peroxidase using the ABC method (Vector laboratories). Detection was carried out with the use of diaminobenzidine tetrahydrochloride (DAB) as substrate and enhanced with copper sulfate and nuclei were counterstained with Mayers Hematoxylin. After the staining samples were dehydrated with alcohol and xylol before mounting in Permount.

### Statistics

Fisher’s exact test was used to compare the frequency of the *SEC63* c.1703_1705delAAG variant in DNA samples from PCLD patients with healthy controls, and to compare the presence of LOH and somatic point mutations in cyst between patients with a *PRKCSH* or a *SEC63* germline mutation. The 95% confidence interval of the proportion of cysts with LOH was calculated with the Newcombe-Wilson method for proportions without continuity correction [18].

## Results

### Patient Tissue Samples

We obtained tissue samples from three patients with a known *SEC63* germline mutation (Table 1), these 3 patients are unrelated and no affected family members are known. We also screened genomic DNA for mutations in *PRKCSH*, the other gene involved in PCLD, to exclude additional germline mutations.

**Table 1:** Somatic mutation analysis.

	Age*	Sex	Tissue samples	Heterozygous germline mutation	# of cysts analyzed	Cysts withLOH
**Patient 1**	38	F	**Frozen**	*SEC63* c. 1703_1705delAAG	34	0 (0%)
**Patient 2**	41	F	**Frozen**	*SEC63* c. 1703_1705delAAG	4	0 (0%)
**Patient 3**	33	F	**Formalin fixed**	*SEC63* c. 958G>T	14	1 (7%)

* Age at time of surgery.

### Somatic Mutation Analysis

**Figure 1:**
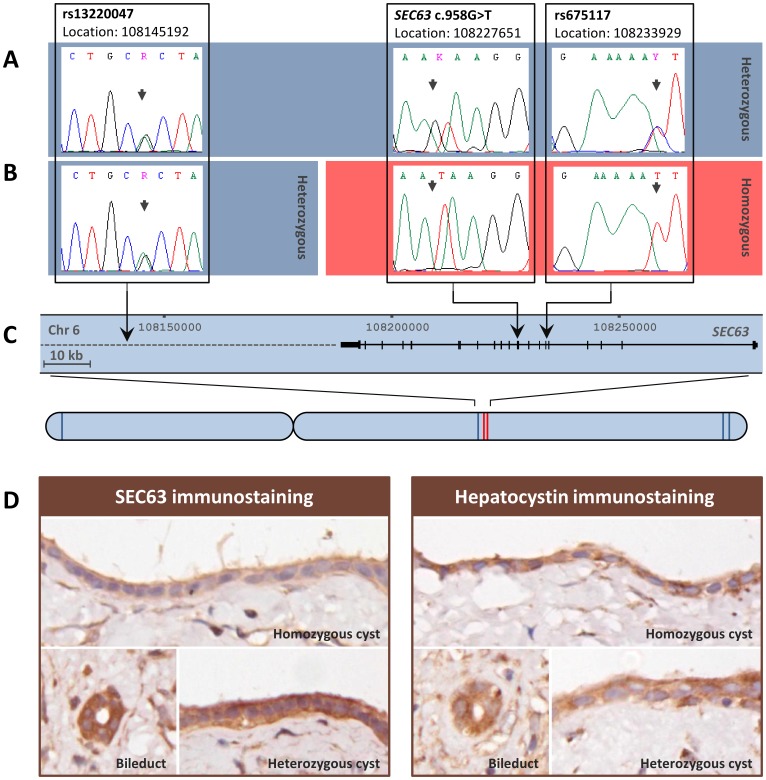
Cyst with loss of heterozygosity. Electropherograms showing the heterozygous germline mutation *SEC63* c.958G>T and two neighbouring SNPs in control liver tissue (A) and in the homozygous liver cyst (B) of patient 3, indicating loss of heterozygosity at *SEC63* c.958G>T and SNP rs675117 in the cyst. The relative locations of the different SNPs are shown, with a close up of the genomic region surrounding the germline mutation (C). The SNPs which remain heterozygous in the cyst with LOH are depicted in blue, SNPs with LOH are depicted in red on the chromosome (C). Immunohistochemical analysis shows the expression of SEC63 and hepatocystin in a normal bile duct, a heterozygous liver cysts and the homozygous liver cyst with LOH at SEC63, in a paraffin-embedded formalin-fixed tissue section of patient #3 (D).

Using laser microdissection we collected epithelial cells from 52 cysts and found LOH to be present in one cyst from patient #3 (Table 1, Figure 1B). Next, we screened the coding sequence of *SEC63* and *PRKCSH* by Sanger sequencing in the remaining cysts of patient#1 and patient #2, no somatic mutations were present. Due to the formalin fixation in the samples of patient #3 we could not sequence *SEC63* and *PRKCSH* to detect other somatic mutations in these samples.

### LOH Region Analysis

We used 5 informative (heterozygous) single nucleotide polymorphisms (SNPs) across chromosome 6 to determine the extent of the region with LOH (Figure 1A, B and C). This demonstrated that the LOH region extends over 6 kb from the site of the germline mutation *SEC63* c.958G>T (in exon10) to exon 6 in the same gene. The SNP (rs13220047) located at a distance of 82 kb remains heterozygous indicating that a genomic breakpoint occurred within this region. In tumour samples LOH regions are often telomeric [19], [20] and extent from one breakpoint towards the end of the chromosome. This was not the case in our sample, where the telomeric SNPs remained heterozygous (Figure 1C).

### Immunohistochemistry

Immunostaining of SEC63 and hepatocystin showed that the hepatocystin staining was similar between cysts, whereas for SEC63 the intensity of the staining was reduced in the cyst with LOH but appears normal in the cysts without loss of heterozygosity (Figure 1D).

### Somatic Mutations Only Present in Patient with Severe Germline Mutation

We found LOH in one of the 14 cysts of patient #3, while somatic mutations were conspicuously absent from samples from the other 2 patients (38 cysts). We reviewed the different underlying *SEC63* germline mutations and found that the germline mutation of patient #3 is potentially much more severe than those of the other 2 patients. Both patient #1 and #2 carry a heterozygous *SEC63* c.1703_1705delAAG mutation which will lead to an in-frame deletion of a single amino acid: glutamate at position 568 in the protein. In contrast, the germline mutation of patient #3 (*SEC63* c.958G>T) results in a premature stop codon after 319 amino acids (p.Glu320X), which deletes 441 out of 760 amino acids.

**Figure 2:**
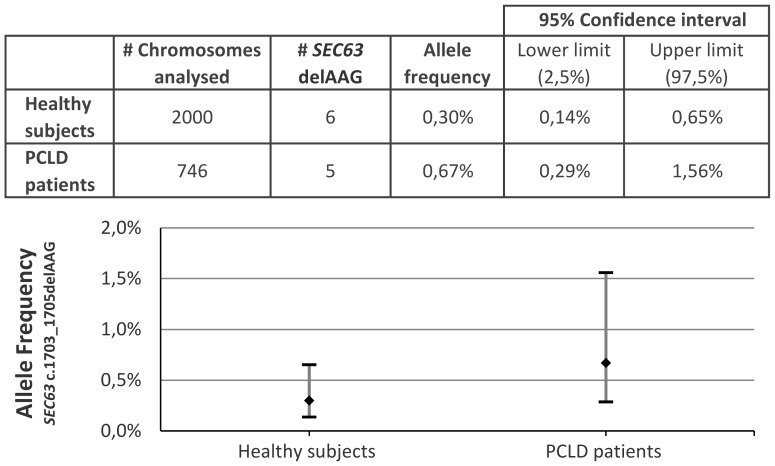
Allele frequency of *SEC63* c.1703_1705delAAG. Allele frequency and confidence interval of the *SEC63* c.1703_1705delAAG gene variant in genomic DNA from PCLD patients and healthy controls.

To determine whether the *SEC63* c.1703_1705delAAG mutation is a true pathogenic mutation or represents a rare (benign) polymorphism, we determined the frequency of this mutation in the normal population by high resolution melting analysis of this region in genomic DNA of 1000 healthy subjects. We found this specific amino acid deletion in 6 out of 2000 normal chromosomes (0,30%), which is similar to the frequency present in a sample of 373 PCLD patients (5/746 chromosomes, 0,67%) as depicted in Figure 2. The difference between both groups was not statistical different (p = 0.18). This suggest that the genomic variant *SEC63* c.1703_1705delAAG is not a pathogenic mutation causing PCLD which may explain the absence of any somatic mutations in this gene.

## Discussion

Our data show that somatic second-hit mutations do occur in *SEC63* mutation carriers with PCLD, which is in line with the second-hit model for disease pathogenesis.

We did not detect somatic mutations in two out of three patients and we hypothesised this was related to the nature of the germline mutation. After reviewing the germline mutations we found that the allele frequency of the *SEC63* c.1703_1705delAAG mutation did not differ between our PCLD patients and the healthy population. This indicates this variant is a (rare) polymorphism which is not associated with PCLD. The literature reports this mutation several times in relation to PCLD [4], [5] but segregation in a family with PCLD has never been shown. Davila et al. reported that they did not find this variant in 360 healthy chromosomes [4], which can be explained by the low allele frequency of this variant. We therefore believe that patients 1 and 2 do not have a pathogenic *SEC63* mutation, but belong to the cohort of PCLD patients in which the genetic cause is still unknown. This is important to know as currently patients with PCLD are being screened, classified and advised based on wrong information.

As *PRKCSH* and *SEC63* are ubiquitously expressed in the human body, it remains unclear why germline mutations would lead to a liver specific disorder. The gene product of *SEC63*, SEC63, is thought to play a role in protein transport across the endoplasmic reticulum (ER) membrane, whereas *PRKCSH* codes for hepatocystin and is part of a heterodimer complex involved in folding of glycoproteins in the ER [21], [22]. However, targeted inactivation of both *PRKCSH* and *SEC63* in an ADPKD mouse model leads to a synergistic increase in disease severity, which suggests that these genes share a biological pathway [23].

Although the frequency of somatic second-hit mutations varies between different genes and genetic disorders, the difference in somatic mutations between cysts from *PRKCSH* patients and this *SEC63* patient is remarkable. We found LOH in only 7% (1/14) of *SEC63* mutated cysts, whereas in *PRKCSH* germline carriers the majority of cysts (76%) acquired LOH [6]. Although the numbers are low, this difference is statistically significant (p<0,00001) and could indicate that these two genes have a different susceptibility to somatic LOH. The immunohistochemical data confirmed loss of SEC63 protein in the cysts with LOH, which indicates that the truncated gene transcript from the mutated *SEC63* c.958G>T allele may no longer be translated into protein or is not recognised by the antibody.

Our study was restricted by the limited amount of available patient tissue. However, we identified and collected all known PCLD liver tissue samples that had been stored within the Netherlands. We obtained samples from three patients, but only one patient carried a bonafide pathogenic *SEC63* germline mutation. Furthermore, the available tissue was formalin fixed which was not a problem for the laser microdissection procedure, but did reduce the yield of the DNA isolation. We were able to get a clear read on the LOH status of all samples, but the sequence efficiency was affected in material derived from formalin-fixed paraffin-embedded tissue. Therefore there may still be presence of, yet unidentified, somatic mutations in these samples.

There is a body of evidence emanating from recent studies supporting the concept of somatic mutations as part of the genetic pathogenesis of benign and malignant disorders. It was recently shown that hamartomata that are part of the Proteus syndrome arise from somatic activating mutations in oncogene *AKT1* [24]. In patients with acute lymphoblastic leukemia and cervical cancer somatic mutations play an important role in the development and prognosis of the disease [25], [26]. Lastly, recurrent somatically acquired mutations of the *SF3B1* gene can be demonstrated in subtypes of myelodysplastic syndromes in which ring sideroblasts are a prominent feature [27]. Collectively, these data demonstrate that genetic inactivation through somatically acquired mutations help to understand the tissue specificity of certain malignant but also non-malignant disorders.

In conclusion, we have now shown that in both *PRKCSH* and *SEC63* somatic second-hit mutations can occur which supports the notion that somatic second-hit mutations are part of the genetic mechanism in cyst formation in PCLD.
